# Functionalization of Plastic Parts by Replication of Variable Pitch Laser-Induced Periodic Surface Structures

**DOI:** 10.3390/mi11040429

**Published:** 2020-04-20

**Authors:** Leonardo Piccolo, Marco Sorgato, Afif Batal, Stefan Dimov, Giovanni Lucchetta, Davide Masato

**Affiliations:** 1 Department of Plastics Engineering, University of Massachusetts Lowell, Lowell, MA 01854, USA; Leonardo.piccolo@phd.unipd.it; 2 Department of Industrial Engineering, University of Padova, 35131 Padova, Italy; Marco.sorgato@unipd.it (M.S.); giovanni.lucchetta@unipd.it (G.L.); 3 Department of Mechanical Engineering, University of Birmingham, Birmingham B15 2TT, UK; bxa361@student.bham.ac.uk (A.B.); s.s.dimov@bham.ac.uk (S.D.)

**Keywords:** laser-induced periodical surface structures, micro-injection molding, replication, surface wettability

## Abstract

Surface functionalization of plastic parts has been studied and developed for several applications. However, demand for the development of reliable and profitable manufacturing strategies is still high. Here we develop and characterize a new process chain for the versatile and cost-effective production of sub-micron textured plastic parts using laser ablation. The study includes the generation of different sub-micron structures on the surface of a mold using femtosecond laser ablation and vario-thermal micro-injection molding. The manufactured parts and their surfaces are characterized in consideration of polymer replication and wetting behavior. The results of the static contact angle measurements show that replicated Laser-Induced Periodic Surface Structures (LIPSSs) always increase the hydrophobicity of plastic parts. A maximum contact angle increase of 20% was found by optimizing the manufacturing thermal boundary conditions. The wetting behavior is linked to the transition from a Wenzel to Cassie–Baxter state, and is crucial in optimizing the injection molding cycle time.

## 1. Introduction

Polymers are materials characterized by very different properties that can be tailored for many applications [1]. Engineers are constantly trying to enhance their desired properties or to straighten the weaknesses of these materials. Surface functionalization has emerged as a solution to the needs of various stakeholders. Functional surfaces find application as self-cleaning [2,3,4], scaffolds [5,6], tissues [7,8,9], optical parts [10], anti-icing [11,12], in friction reduction [13,14], and in engineering of injection mold surfaces [15]. All these applications generated an increasing demand to develop reliable and cost-effective mass manufacturing technologies for polymer surface replication. 

Functionalization of polymer surfaces can be obtained by modifying their chemistry (e.g., using coatings) or through topographic modifications (e.g., texturing) [16]. The chemical modification of a surface typically requires an additional step on the production of the part. This paper focuses on tailoring the surface properties of plastic parts through topographic modification, exploiting a net shape process. Functionalization can be described by considering the wettability of a surface [17]. Wettability is a fundamental characteristic of solids, and is governed by surface chemistry and morphology. It measures the ability of a liquid to maintain contact with a solid surface, and is determined by the balance between cohesive (inside the liquid) and adhesive (between liquid and the solid surface) forces. By changing the surface topography, adhesive interactions can be modified. Wettability can be characterized by utilizing contact angle measurements. A hydrophilic surface exhibits a contact angle smaller than 90°. Conversely, a hydrophobic surface yields a contact angle above 90°. When the contact angle exceeds 150°, the surface is defined as super-hydrophobic. 

When considering surface topographies, two models were developed to explain the interaction between asperities and liquid droplets. The Wenzel state assumes that liquid droplets follow the asperities of the surface, emphasizing the intrinsic hydrophobic or hydrophilic behavior of the material [18]. The Cassie–Baxter state predicts that asperities can trap air bubbles inside them, resulting in an inhomogeneous surface [19]. When the fraction of a solid in contact with a water droplet decrease, it means that more air is trapped, and thus the contact angle increases. The Wenzel state depends directly on surface roughness, the Cassie–Baxter state depends on the wet fraction of the solid. The aim of surface structuring is to create patterns that optimize surface roughness and minimize the solid fraction of a surface in contact with the liquid.

Texturing of plastic parts can be achieved either via direct patterning or by replicating the surface of a tool. The former approach is slow, expensive and may lead to some chemical changes on the surface [20,21], while the latter offers more efficient manufacturing opportunities. Indeed, the high investment costs of patterning the mold can be adequately justified when the tool is used for long production runs. Mold texturing technologies can be divided into a top-down (i.e., removing material) and a bottom-up (adding material) approach [22]. Top-down technologies are approaches that shape the pattern by removing material from the substrate using either hard tools (e.g., milling, etching) or energy (e.g., laser engraving, electrical discharge machining). Bottom-up technologies exploit self-assembly or self-organization properties of certain materials to create small (i.e., up to few nanometers) and accurate structures. The investment and operating costs of top-down techniques are high, and there are substantial limitations on the patterning of large areas and complex surfaces. At the same time, despite their easier scalability, bottom-up technologies are characterized by the worst capabilities in terms of geometry, and feature size is limited [23].

Mold texturing for injection molding applications requires a high speed and flexibility [24]. Laser patterning has been proven effective for the fast-engraving of highly uniform micro-structured surfaces on a large variety of materials, including steel [25,26,27]. Furthermore, throughputs as high as 1 cm^2^ per 10 s have been reported for metals when producing self-organized sub-micron structures [28]. High texturing speeds characterize this type of surface modification due to the unique structuring phenomena happening on the surface. The laser beam irradiates the surface on an area that is much bigger than the feature size, leaving a sort of fingerprint characteristic of the particular laser beam. Femtosecond lasers, typically exploited for the formation of Laser-Induced Periodic Surface Structures (LIPSSs), are pulsed lasers characterized by extremely short pulse durations and high peak power. The interference pattern formed by the incident wave (i.e., the laser light wave) and the exited Surface Electromagnetic Wave (SEW) rule the patterning phenomena. The repeated interference between the two waves creates a pattern of energy distribution that ionizes and melts the very first layer of material on the surface, accordingly creating the typical ripples of the LIPSS pattern [29].

The morphology of the LIPSS pattern can be modified by changing the laser setup. The regularity and depth of the structures can be optimized, controlling the way the beam scans the surface and the beam fluence (i.e., energy delivered on a specific area). The pitch between consecutive structures is strongly linked to the wavelength of the laser light (typically, the pitch is slightly smaller than the laser wavelength [30]).

Tunable laser systems (variable beam wavelength) are more expensive than their single-frequency counterparts due to the added complexity, while their output power is generally compromised. Therefore, other approaches can be considered to modify LIPSS periodicity. Varying the laser Beam Incident Angle (*BIA*) relative to the surface can be one method for varying the ripples’ pitch. The phenomenon is controlled by the energy interference pattern formed by the incident wave and the exited Surface Electromagnetic Wave (SEW). This interference may result in a different period of the pattern or a pattern with two periods. For a Transverse Magnetic (TM) wave, the period of the structures is given by

(1) $$\Lambda_{1,2}=\frac{\lambda }{1\pm \sin\theta }$$

where *Λ* is the pattern pitch, *λ* is the laser wavelength and *θ* the angle between the laser beam and the normal surface direction [31].

Hot embossing and injection molding are the most efficient technologies to achieve replication of submicron-structured plastic parts [32]. However, when it is required to apply textures on 3D geometries, only micro injection molding (μIM) can be considered [33]. The replication of submicron-structured surfaces is challenging since the injected molten polymer tends to solidify quickly touching the cold mold, hindering filling of the micro-cavities [34]. Replication is highly influenced by process parameters (mold temperature, melt temperature, injection velocity and holding pressure) and mold design (e.g., distance of the patterned area from the gate and part thickness) [34,35]. In the literature, many experimental studies have focused on the optimization of the μIM process and auxiliaries to overcome the manufacturing issues driven by the replication of high aspect ratio structures [36,37]. Rapid Heat Cycle Molding (RHCM) is commonly used to increase mold temperatures above the polymer glass transition temperature (*T*_g_) during the injection phase. The fast cooling of the mold after filling allows the ejection of the part [38]. However, RHCM requires significant investments, and the cycle time is highly affected.

The replication performance of the process is generally evaluated in terms of surface topography without considering its functionality. In this work, the functionalization of the surface is characterized considering its wetting properties. The correlation between replication and wettability is not clear yet. With functionalization of the surface being the aim of the whole process, understanding the roles of the process features on the wetting phenomena can drive process optimization and productivity.

In this work, the process chain for manufacturing functional submicron-structured plastic surfaces is studied. A femtosecond laser source is used to generate different submicron structures on the mold surface. The laser ablation process is characterized and studied to understand the effect of its parameters on ripple morphology. The mold surface textures are then replicated by the injection molding of different thermoplastic resins. Texture design, laser processing and the wettability of parts are studied holistically to optimize the manufacturing process as well as the functionality of the parts.

## 2. Materials and Methods 

### 2.1. Process Chain and Experimental Approach

**Figure 1:**
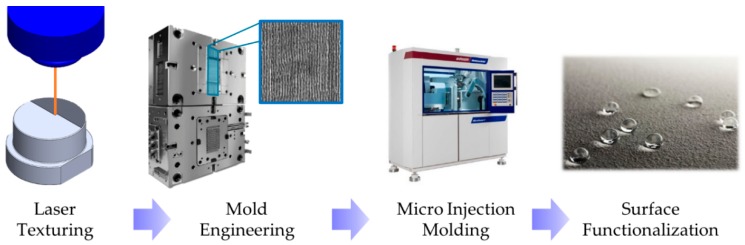
Schematic of the process chain utilized in this work to functionalize plastic parts.

In this work, the process for cost-effective and fast production of functionalized plastic parts using a femtosecond laser is presented and characterized. The proposed process chain accomplishes the plastic part functionalization by laser texturing of the mold surface and replication by μIM (Figure 1). The sub-micrometric pattern on the conventional steel surface is engraved using an ultrafast pulsed laser. The texture replication is accomplished using µIM, since it is a suitable process to replicate even small features on 3D parts with a wide range of thermoplastic materials at a low cycle time. Texturing of plastic parts leads to improved wetting properties.

The process chain was validated by texturing four mold inserts with different laser process conditions. The µIM process capability to replicate the surface structures was experimentally investigated for different temperature boundary conditions. As the mold surface temperature decreased, the melt cooling rate increased, leading to higher viscosities and preventing the replication of the submicron structures. The process performance was evaluated considering the functionality of the manufactured plastic parts. The correlation between surface functionalization and polymer replication is discussed comparing the plastic surface wetting behavior with the replicated topographies. 

### 2.2. Mold Design

**Figure 2:**
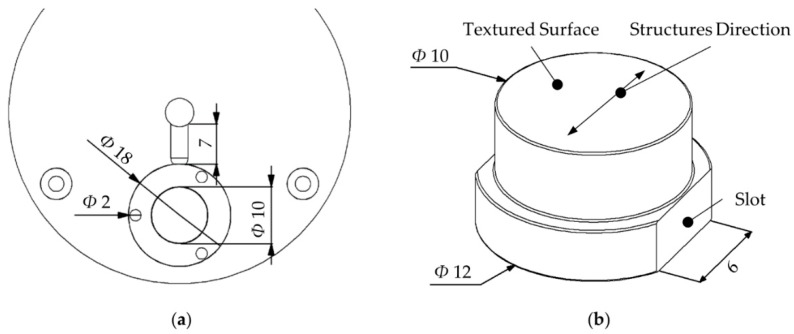
(**a**) Design of the mold cavity. (**b**) Design of the textured insert and alignment features. (All dimensions are in millimeters.)

The plastic part designed for this study was a disk with a diameter of 18 mm and a wall thickness of 1.5 mm. The cavity was placed on the B-side of the mold at the end of a 7-mm long semi-circular cold runner with a radius of 1.5 mm (Figure 2a). The textured surface had a diameter of 10 mm and was assembled in the cavity using round inserts (Figure 2b). Two alignment slots were machined on the side of the insert. They were used to align the surface structures with the polymer flow. Ejector pins were evenly distributed around the cavity to ensure uniform demolding force on the molded plastic part. Electrical heating was combined with water cooling for the RHCM process and to control the mold surface temperature.

### 2.3. Mold Surface Treatments

Five mold inserts were machined and polished to obtain a smooth surface finish (*Sa* below 25 nm) before the generation of the submicron structures by laser ablation. Then, the surface of the inserts was treated with ultrashort laser patterning using a femtosecond laser source (Satsuma, Amplitude Systems, Pessac, France). The laser process parameters, reported in Table 1, were selected based on a previous optimization study to obtain a uniform pitch and depth of the ripples [39]. The laser beam polarization was unchanged during the trials, and the inserts were oriented identically in the Computer Numerical Control (CNC) machining setup, ensuring the same ripples’ directions over the four textured samples. The irradiation was done in such a way ensuring the beam p-type linear polarization was perpendicular to the scanning direction. Laser patterning was carried out by focusing the laser beam using a telecentric lens down to a diameter (2*w*_0_) of 40 µm and moving it with a galvanometer scanner over the insert surface at a speed of (*v*) 1500 mm/s with a pulse frequency (*f*) of 250 kHz. Thus, the pulse distance (*L*) was 6 µm (distance between two consecutive pulses). The same value was used for the scanning step over distance (*H*) (spacing between two consecutive scanning lines), thus obtaining the same pulse overlaps in both X and Y directions. 

The pitch distance between consecutive ripples was changed in different inserts by changing the beam incidence angle (*BIA*). *BIA* values were changed between 0° (normal incidence) and 30° while keeping the polarization parallel to the plane of incidence. In particular, the four inserts A, B, C and D were produced with *BIA*s of 0°, 10°, 20°, and 30°, respectively. The pulse fluence is dependent on the beam profile on the insert surface, and therefore it is slightly reduced as the beam area increases when the *BIA* value is increased. When the surface is tilted the beam spot can be assumed to be an ellipse with the axis size a trigonometric function of *BIA* [40]. Thus, the respective pulse fluence (*F*) values for *BIA*s 10°, 20° and 30° are 197, 188 and 174 mJ/cm^2^, respectively. 

### 2.4. Surface Characterization

The roughness of the polished inserts was measured before laser patterning using a confocal profilometer (3D S Neox, Sensofar, Barcelona, Spain). The instrument was used in confocal mode with a 100X lens, and surface roughness (*Sa*) of the inserts was evaluated.

The morphology of the sub-microstructures on the mold insert was characterized using Scanning Electron Microscopy (SEM - FEI, Quanta 400, Thermo Fisher Scientific, Hillsboro, OR, USA). This technique permits the homogeneity of the texture of the entire surface to be quickly examined. 

The geometrical characterization of the ripples was carried out using Atomic Force Microscopy (AFM –CP II, Veeco Digital Instruments, Bruker, Billerica, MA, USA). This first morphological characterization is needed to prove the representativeness of the small AFM scanned area. The AFM analysis (Table 2) was repeated onto two 10- × 10-μm areas over each insert. The mean height of the structures was evaluated with the analysis of the acquired point clouds exploiting the Abbott-Firestone curves method according to the standard DS/EN ISO 25178-2 [41]. The material ratio curve (i.e., Abbott-Firestone curve) describes the increase of the material portion of the surface with increasing the roughness depth. For each acquired topography, the core surface roughness was determined from the linear representation of the material ratio curve and addressed to the mean topographic height.

The structure periodicity was carried out using 2D FFT (2D Fast Fourier Transform) analysis of the entire measured surface. The main peaks of the spectrum can be correlated to surface periodicities [42].

**Figure 3:**
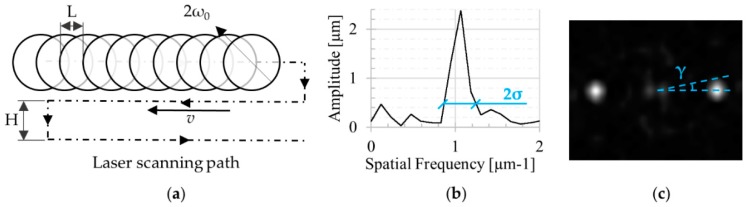
(**a**) Scanning path for the laser surface structuring (clarity dimensions are not correctly scaled); (**b**) FWMH (Full Width at Half Maximum) evaluation by the horizontal profile extracted from the 2D FFT of the AFM point clouds; (**c**) representative 2D FFT micrographs describing the dispersion angle.

Two parameters were considered to describe the pattern quality, (i) regularity (*R*), and (ii) homogeneity. Pattern regularity refers to the uniformity of the pitch distance between consecutive structures. This parameter was evaluated from the 2D FFT analysis of the topographies acquired using AFM. The regularity is represented by the standard deviation of the normal distribution associated with the peak of the considered ripple periodicity. Since the pitch variation was within the direction of the ripples, a horizontal profile of the 2D FFT analysis was extracted (Figure 3b). The regularity of the pattern was evaluated considering the ratio between the standard deviation (*σ*) of the normal distribution associated with each pitch (*Λ*) present along with the pattern and the pitch itself:

(2) $$R=1-\overset{n}{\underset{i=1}{\sum }}\frac{2\sigma_{i}}{\Lambda_{i}}$$

The homogeneity of the texture was analyzed considering the ripple dispersion angle *γ* (i.e., the angle deviation of the ripples from the straight parallel configuration). This dispersion angle can be adequately described considering the angle *γ* that encloses the areal 2D FFT peak dispersion of the ripple frequency (Figure 3c).

The same morphologic and topographic analyses were performed on the plastic part surfaces to investigate the replication accuracy. SEM analysis was carried to examine the homogeneity of the replication over the entire surface. The AFM setup was adapted for the measurements on the plastic parts to optimize accuracy (Table 2).

### 2.5. Materials and Manufacturing System

A commercial polystyrene (PS Crystal 1540, Total Petrochemicals & Refining, Bruxelles, Belgium) and a poly-methyl methacrylate (PMMA, Polycasa Acryl G55, 3A Composites, Statesville, NC, USA) were selected for the experimental work. Their main properties are reported in Table 3. These polymers are commonly used in µIM due to their high flowability, high optical clarity, high transparency, and high impact strength.

A state-of-the-art micro injection molding machine (MicroPower 15, Wittmann-Battenfeld GmbH, Kottingbrunn, Austria) was used for the μIM experiments. The accuracy and repeatability of the injection phase were guaranteed by the separation of the metering (14 mm screw) and injection (5 mm plunger) units. A maximum injection pressure of 2700 bar was used as the limit during the molding cycles. The mold temperature was varied from 40 to 120 °C, and a 10 °C gap was chosen between the tested temperatures. The other process parameters of the injection molding for each material were kept constant (Table 4). To ensure the stability of the process, 20 molding cycles were performed before the first part was collected. For each condition, 15 parts were sampled.

### 2.6. Wetting Characterization

**Figure 4:**
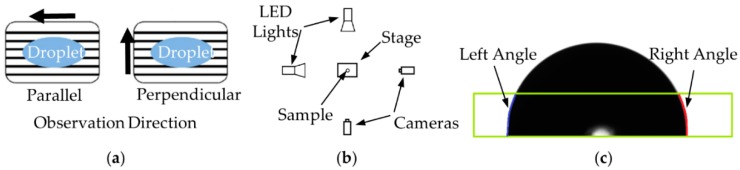
(**a**) Observation direction of the water droplets on the textures surface; (**b**) layout of the wetting test setup; (**c**) estimation of the contact angles through the fitting of the droplet shape. The green square defines the searching area that the software keeps.

The wetting properties of the textured plastic surface were evaluated from contact angle measurements and benchmarked against those of a smooth plastic surface. The equipment used for the measurements consisted of a horizontal stage, a motor-driven micrometer syringe (UMP3) with a needle (diameter 0.21 mm), two background illumination sources (LED Pholox) and two cameras (MANTA G-146, Allied Vision Technologies GmbH, Exton, PA, USA) with a telecentric 2X lens (VS-TC2-110, VS Technology, USA) (Figure 4a). The measurements were carried out using water droplets with a total volume of 500 nL. 

The contact angle was measured along two directions at 90° to account for the effect of ripple directionality on the drop shape (Figure 4b). The acquired images were elaborated by fitting the shape of each droplet to calculate the contact angle, as shown in Figure 4c. The wetting characterization was performed on three parts made with each polymer for each combination of processing parameters. On each plastic part, six water droplets were deposited and their contact angles acquired. This allowed for the acquisition of 36 contact angle measurements per experimental condition.

## 3. Results

### 3.1. Generated Mold Topographies

Laser patterning of the 10-mm diameter circle required about 25 s, leading to a surface patterning speed of about 3.3 mm^2^/s. This demonstrates the speed of the ultrafast laser technology and its flexibility. Indeed, scaling up the texturing process would be possible, and processing times would be reasonable, allowing elevated texturing speeds and customization. 

**Figure 5:**
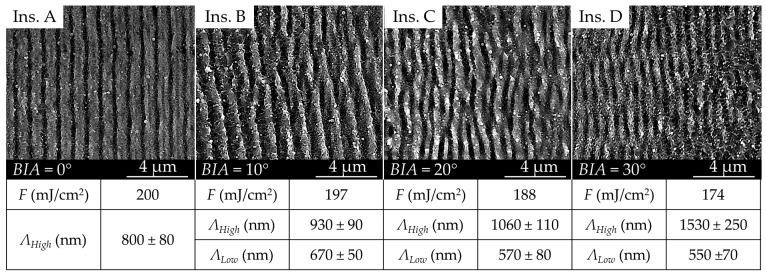
SEM micrographs of the textured surfaces on the steel mold inserts for different beam incidence angles (*BIA*s).

The morphology in terms of homogeneity and regularity of the sub-micrometric structures was initially characterized using SEM. SEM micrographs indicate that the LIPSSs were uniform over the entire insert surface, confirming the reliability of the laser processing at high scan speeds. Considering the SEM pictures in Figure 5, changes in the ripples’ morphology could be observed when increasing the *BIA* value. In particular, the ripples’ pitch and overall regularity appeared to decrease as the *BIA* increased. The periodicity of the LIPSSs was varied by changing the angle of incidence, and a second period was created in the pattern. The appearance of a second period proves that beam tilting is an effective method to change the surface texture morphology.

The AFM analysis allowed a quantitative characterization of the inserts’ topographies; in particular, the pitch distance between consecutive ripples and the homogeneity of the pattern were evaluated. These parameters were studied by considering the laser beam incidence angle and by defining and plotting some topographical parameters.

**Figure 6:**
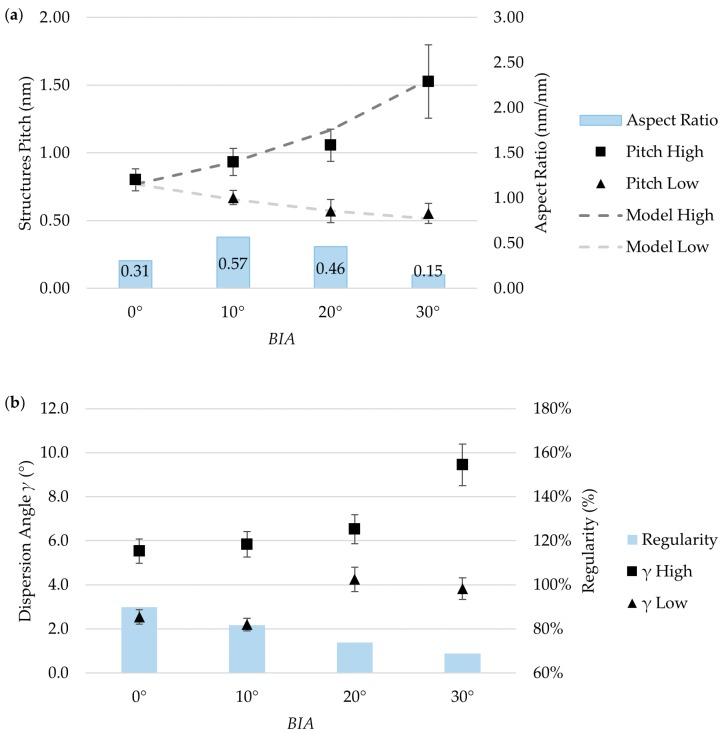
(**a**) Results of the AFM characterization of the four laser-machined steel mold inserts in terms of pitch and aspect ratio of the pattern; (**b**) plot of the pattern homogeneity study results for the four mold inserts.

SEM micrographs suggested that changes in *BIA* could lead to the onset of a secondary periodicity in the ripples pattern. Here, we call *Λ_high_* the higher (primary) pitch distance and *Λ_low_* the smaller (secondary) pitch distance. The pitch distance for the structures can be calculated for different tilting angles as follows:

(3) $$\Lambda_{Low,High}=\frac{\varepsilon \cdot \lambda }{1\pm \sin\left(BIA\right)}$$

where *λ* is the laser wavelength, and *ε* is a loss factor defined to consider the wavelength-to-pitch ratio. In fact, when the beam was normalized to the workpiece surface (i.e., *BIA* = 0°), a pitch of 770 nm was measured, which is slightly lower than the laser wavelength (i.e., 1030 nm). To account for this phenomenon, a loss factor ε = *Λ*_α_ = 90°/*λ* was introduced in the theoretical model [31]. The experimental data are plotted in Figure 6a against the model defined in Equation (3 to further understand the ripple periodicity. It can be observed that the difference between the two periods became larger as the *BIA* increased. This suggests the possibility of modifying surface functionality and potentially generating hierarchic textures. The influence of *BIA* on structures’ aspect ratios has not yet reported in the literature. However, an initial increase then decrease of the aspect ratio was observed with the *BIA* increased, as shown in Figure 6a. Figure 6b shows the effect of *BIA*s on ripple quality and homogeneity. It was observed that pattern regularity decreases as the *BIA* increases. This is probably related to the interference phenomena occurring on the surface during the short pulse irradiation durations, and the formation of irregular intermediary ripples in-between the primary periodic structures. This trend has also been confirmed by the dispersion angle γ.

The effects of *BIA* on both pattern regularity and homogeneity can also be evaluated using SEM micrographs (Figure 5). The pattern regularity describes how consistent the ripples’ periodicity was, whereas the dispersion angle was linked to their propagation direction. Variations in pattern periodicity within a sample translated to a decrease in *R*, while the formation of intermediary ripples that bifurcated and intertwinded resulting in higher dispersion angle γ (Figure 6b).

Considering the results of the metrological characterization carried out on the different textures, Insert B was selected for the injection molding experimental campaign. Indeed, at *BIA* = 10°, the ripples presented the best compromise, exhibiting both a high aspect ratio and homogeneity while displaying a low dispersion angle value. Insert B was selected for the polymer replication studies because its topography was characterized by the highest aspect ratio, which is well-known as the key parameter to controlling the replication of textured surfaces [43].

### 3.2. Sub-Micron Structures Polymer Replication

**Figure 7:**
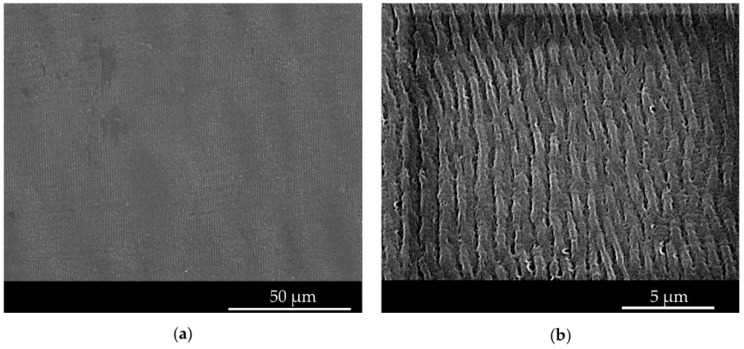
SEM images of the ripples replicated on PS samples. (**a**) Low magnification SEM picture of a PS sample molded at a mold temperature of 50 °C; (**b**) high magnification pictures of PS molded at 50 °C.

The surface of the molded plastic parts was initially characterized using SEM. The SEM analysis showed that the quality of the replicated ripples was homogenous along the textured surface for both polymers and for different mold temperatures (Figure 7). Micrographs show the absence of significant defects or areas that are not homogeneously replicated.

**Figure 8:**
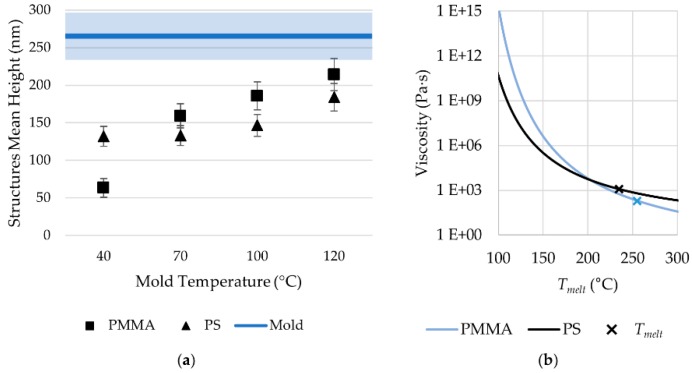
(**a**) Replicated structure heights for PMMA and PS. (**b**) Zero shear viscosity dependence on temperature for PMMA and PS.

The AFM scans were processed using the Abbott-Firestone curves method. Core height (*S_k_*) was evaluated for the different topographies and compared to mold measurements. Figure 8a shows the replication performance of the two resins, indicating the difference between the replicated aspect ratio and that of the mold. Moreover, the effect of mold temperature was analyzed, indicating that, as expected, higher mold temperatures lead to higher replications. However, the effect of mold temperature was different for the two resins. When molding PMMA, the effect of increasing mold surface temperature was more significant than for PS. This could be related to the temperature dependency of PMMA polymer melt viscosity. In fact, as the hot melt touches the cold mold, its viscosity increases significantly. This viscosity rises strongly depending on the polymer/mold thermal boundary and polymer properties. Figure 8b compares the Newtonian viscosity for the two polymers as a function of temperature. The behavior of the two polymers intersects around 210 °C, which is below melt temperature. Thus, during molding, PMMA has a higher melt viscosity than PS. This explains the smaller replication obtained when using PMMA and the higher sensitivity to an increase of mold temperature. 

### 3.3. Plastic Parts Surface Wettability

**Figure 9:**
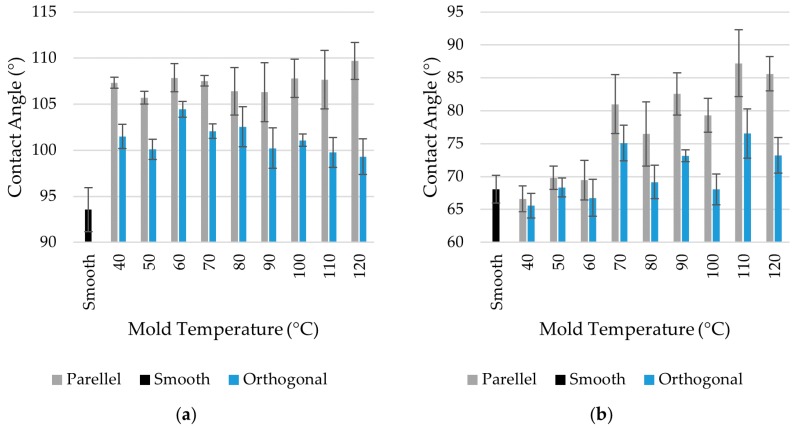
Results of contact angle measurements for (**a**) PS and (**b**) PMMA plastic parts.

The analysis of the contact angles indicates that the wetting properties of plastic parts were affected by the replicated sub-micron structures. Due to the anisotropy of the textured surface, the drop has an ellipsoidal shape. To account for this effect, the contact angle along two directions (see Figure 4) was measured. The results reported in Figure 9 show that the parallel angle assumed higher values than the perpendicular one, indicating that water drops spread easier along the ripples. The maximum water contact angle increase was about 20% for PMMA and 17% for PS with respect to the smooth part.

The two polymers showed different wetting properties as a function of the mold temperature. Indeed, molding PMMA at a higher temperature substantially improved the functionalization of the surface. In particular, the most significant contact angle increase (i.e., 17%) was observed when increasing the temperature from 60 to 70 °C. This allowed the identification of a threshold value above which increasing the mold temperature did not yield significant variations in part functionality. Conversely, the wetting properties of the PS molded part did not indicate any substantial effect of the mold temperature in the investigated range.

### 3.4. Comparison between Achieved Replication and Functionalization

**Figure 10:**
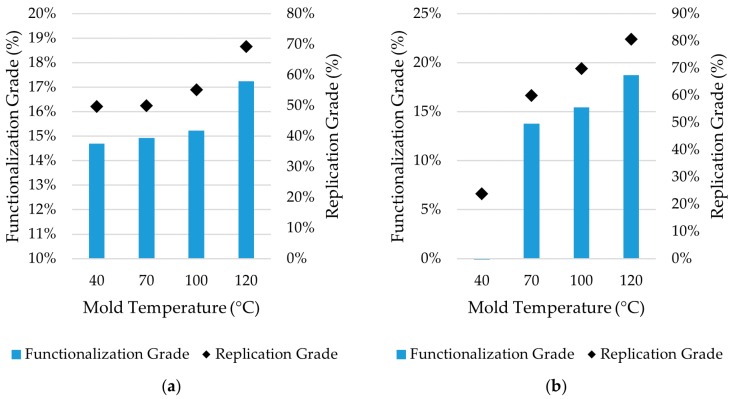
Comparison between polymer surface replication grade and functionalization grade for (**a**) PS parts and (**b**) PMMA parts.

The plot shown in Figure 10 compares the polymer replication grade of submicron structures and the functionalization grade. The replication grade expresses the ratio between the height of the structures on the polymer part and the depth of the mold structures. The functionalization grade describes the contact angle gain for the textured plastic parts with respect to the smooth ones. For parts molded with PS, the grade of functionalization and replication follows the same trend, showing similar results for all tested mold temperature values. Conversely, when molding PMMA at low mold temperature, the higher melt viscosity leads to lower replication. In this condition, water droplets have similar behavior as when they were deposited onto a smooth surface. The surface was not yet practically functionalized. As the mold temperature increases, the replication grade increases, and the surface becomes more hydrophobic. The further increase of mold temperature beneath the *T_g_* of the polymers leads to an increase in replication accuracy that is not followed by the wetting properties of the surface. This can be linked to Cassie–Baxter behavior of the water droplet, which is no longer affected by the depth of the structures on the surface. Indeed, in the Cassie–Baxter state the droplet does not follow the surface topography entirely, but the droplet achieves contact only with the top of the asperities.

## 4. Conclusions

In this work, different textures were generated by laser ablation and replicated by injection molding with the goal of functionalizing the surface of plastic parts. The process consisted of the fast fabrication of surface structures on steel using a femtosecond laser ablation and their reproduction on plastic parts by µIM. A mold setup with interchangeable inserts was designed, and two different polymers were tested. Mold inserts were treated with a polarized ultrashort pulsed laser source to obtain highly aligned and uniform LIPSSs. The achieved functionalization of the textured surface was studied as a function of the mold temperature. The molded parts were characterized by measuring the degree of replication and their wetting properties.

The textured mold surfaces confirm the suitability of laser technology for scaling up surface functionalization, and every insert was textured in less than 2 min. Tilting the beam angle on the surface is an effective way to manage the morphology of the sub-micron texture, even inducing the formation of a two-period pattern. A loss on the regularity of the pattern occurred when irradiating the surface with higher angles of beam incidence. Further work would be required for the optimization of laser scanning with a tilted beam.

The results of the µIM experimental tests show that the presence of a pattern increases surface functionality. Maximum water contact angle increases of 20% and 17% were found for PMMA and PS samples, respectively. The results show that the achieved functionalization was affected by the polymer temperature, depending on melt viscosity. When molding polymers with a high viscosity temperature dependence, the hydrophobicity of the surface changes rapidly above a threshold value. This temperature was identified between 60 and 70 °C for PMMA.

Further increases of mold temperature did not affect the hydrophobicity, even if the replication accuracy was higher. This phenomenon can be linked to the Cassie–Baxter behavior of the water droplets, which were no longer affected by the depth of the structures. The results for both polymers is attractive from the perspective of producing functionalized low-cost parts. Indeed, keeping a lower mold temperature leads to a decrease in manufacturing costs both from energy consumption and cycle time savings.

## Figures and Tables

**Table 1:** Laser process parameters.

Parameter	Value
Wavelength (nm)	1030
Pulse duration (fs)	310
Pulse energy (µJ)	2.52
Frequency (*f*) (kHz)	250
Scanning speed (*v*) (mm/s)	1500
Step over (*H*) (µm)	6
Spot diameter (2*w*_0_) (µm)	40
Fluence (*F*) at normal incidence (mJ/cm^2^)	200

**Table 2:** AFM measuring setup for the topography of the plastic parts.

Property	Insert	Part
Tip material	Silicon	Gold
Tip curvature radius (nm)	6	10
Cantilever length (μm)	180	125
Cantilever width (μm)	18	30
Cantilever thickness (μm)	0.6	3
Force constant (N/m)	0.05	1.45
Resonant frequency (kHz)	22	87

**Table 3:** Main properties of the selected polymers.

Property	PS	PMMA
Density (g/cm^3^)	1.05	1.18
Melt flow index (g/10 min)	12 (200 °C-5 kg)	21 (230 °C-3.80 kg)
Glass transition temperature (°C)	100	105
Drying time (h)	2	6
Drying temperature (°C)	70	80
Recommended ejection temperature (°C)	85	85
Recommended mold temperature range (°C)	30–50	40–90

**Table 4:** Main processing conditions chosen for the injection molding experiments with the two resins.

Parameter	PS	PMMA
Melt temperature (°C)	235	255
Back pressure (MPa)	5	2
Switch-over pressure (MPa)	80	82
Injection speed (mm/s)	110	110
Packing pressure (MPa)	45	50
Cooling time (s)	10	10
Mold temperature range (°C)	40–120	40–120
